# Improved blackwater disinfection using potentiodynamic methods with oxidized boron-doped diamond electrodes

**DOI:** 10.1016/j.watres.2018.04.022

**Published:** 2018-09-01

**Authors:** J.O. Thostenson, R. Mourouvin, B.T. Hawkins, E. Ngaboyamahina, K.L. Sellgren, C.B. Parker, M.A. Deshusses, B.R. Stoner, J.T. Glass

**Affiliations:** a Department of Electrical and Computer Engineering, Duke University, Durham, NC, United States; b Center for WaSH-AID, Duke University, Durham, NC, United States; c École Centrale de Lyon, Écully, France; d Research Triangle Institute (RTI) International, Research Triangle Park, NC, United States; e Department of Civil and Environmental Engineering, Duke University, Durham, NC, United States

**Keywords:** Boron-doped diamond, Reactive oxygen species, Electrochemical disinfection, Electrochemical advanced oxidation processes (EAOP), Blackwater, Decentralized water treatment

## Abstract

Electrochemical disinfection (ECD) has become an important blackwater disinfection technology. ECD is a promising solution for the 2 billion people without access to conventional sanitation practices and in areas deficient in basic utilities (e.g., sewers, electricity, waste treatment). Here, we report on the disinfection of blackwater using potential cycling compared to potentiostatic treatment methods in chloride-containing and chloride-free solutions of blackwater (*i.e.*, untreated wastewater containing feces, urine, and flushwater from a toilet). Potentiodynamic treatment is demonstrated to improve disinfection energy efficiency of blackwater by 24% and 124% compared to static oxidation and reduction methods, respectively. The result is shown to be caused by electrochemical advanced oxidation processes (EAOP) and regeneration of sp^2^-surface-bonded carbon functional groups that serve the dual purpose of catalysts and adsorption sites of oxidant intermediates. Following 24 h electrolysis in blackwater, electrode fouling is shown to be minimized by the potential cycling method when compared to equivalent potentiostatic methods. The potential cycling current density is 40% higher than both the static oxidative and reductive methods. This work enhances the understanding of oxygen reduction catalysts using functionalized carbon materials and electrochemical disinfection anodes, both of which have the potential to bring a cost-effective, energy efficient, and practical solution to the problem of disinfecting blackwater.

## Highlights

• Demonstration of improved blackwater disinfection using potentiodynamic methods.
• Improvement is linked to control of electrode surface chemistry.
• A surface chemical mechanism is proposed for different electrolytes.
• Method shown to be far more efficient than static potential methods.

## Graphical abstract

**Figure:**
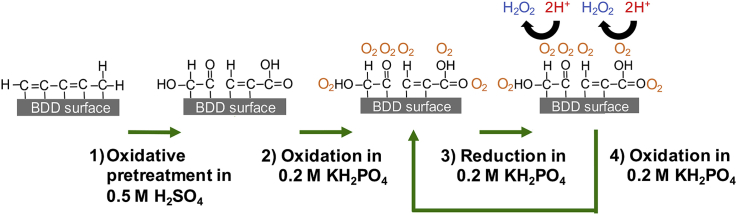

## 1 Introduction

Approximately 40% of the world population does not have access to appropriate sanitation (Water, Sanitation and Hygiene Strategy Overview, 2017). Limited infrastructure and poverty in developing areas has hindered use of suitable blackwater treatment practices. Microbial species such as *E. coli*, helminths, and other excreted pathogens are commonly present in untreated blackwater and lead to a range of illnesses including gastroenteritis. Over 1.4 million people die each year as a result of diarrheal illness caused by poor sanitation or insufficient treatment of wastewater, with 43% of the deaths being children 5 and under (Prüss-Üstün et al., 2008; Wastewater management: A UN-Water Analytical Brief, 2015). While methods such as membrane filtration, UV irradiation, pasteurization, chlorination, and ozonation have been studied for the treatment of wastewater, these treatment practices have seen limited adoption in developing countries due to high investment costs, high energy requirements, complex maintenance, dependence on supply and storage of chemicals, and generation of harmful by-products (Bourrouet et al., 2001; Jefferson et al., 2001; Jhansi et al., 2013; Lazarova, 1999; Rajala et al., 2003).

Electrochemical disinfection (ECD) provides a scalable, low cost, low maintenance, and energy efficient alternative to current disinfection methods (Stoner, 1973; Stoner et al., 1982). Strong oxidants, such as chlorine containing species (CCS) and reactive oxygen species (ROS), can be generated in blackwater without the addition of chemicals. CCS and ROS are effective at inactivating harmful microorganisms such as *E. coli* (Drogui et al., 2001; Gordon et al., 1998; Shimada and Shimahara, 1982; Stoner et al., 1982; Venczel et al., 1997; Venkitanarayanan and Ezeike, 1999). While CCS, such as HClO, Cl_2,_ and ClO^−^, can be efficiently generated and can effectively treat wastewater (Raut et al., 2014, 2013), ROS have been reported to have greater pathogen inactivation efficiency, higher reactivity, and no hazardous long-term effects (Chen et al., 2017; Jeong et al., 2006; Jeong et al., 2009). For instance, H_2_O_2_, an ROS, will decompose into water upon exposure to sunlight. Similarly, ^•^OH, O_2_^−^, and other ROSs will also decompose to water if not used for pathogen inactivation or other reactions. Conversely, CCSs will often linger in solution when unreacted, leading to potentially harmful environmental and physiological side-effects (Background on Drinking Water Standards in the Safe Drinking Water Act (SDWA), 1996; Brungs, 1973).

Several electrode materials have been reported to efficiently produce CCS and ROS. Chlorination of blackwater using CCS is a fundamental method of ECD. Mixed metal oxides (MMOs), BDD, and Pt electrodes have been shown to be effective chlorine generators for ECD at oxidative potentials (Martínez-Huitle et al., 2015; Martínez-Huitle and Ferro, 2006). MMOs have been a focus of ECD (Chen, 2004; Jeong et al., 2009). However, the electrochemical properties of these materials, such as electroactivity and low over-potentials for water electrolysis, lead to oxidant production with diminished coulombic efficiency (Chen, 2004; Jeong et al., 2009). Boron-doped diamond (BDD) is a potential electrode material that does not have these deficiencies. Previous papers have shown that BDD electrodes have low electroactivity, a wide solvent window, mechanical robustness, resistance to corrosion in challenging chemical environments, and ability for polarity reversal without degrading the performance of the electrode (Einaga, 2010; Einaga et al., 2014; Fujishima et al., 2005; Luong et al., 2009; Macpherson, 2015; Marselli et al., 2003; Swain et al., 1998). Application of BDD as electrochemical electrodes to generate CCS and ROS in aqueous environments has also been studied (Jeong et al., 2007, 2006; Jeong et al., 2009). Particularly important is the ability of BDD to generate ROS with improved efficiency over other electrode materials due to the large over-potential needed for water splitting (Cañizares et al., 2002). Less expensive and non-sp^3^ carbon-based materials, such as activated carbon or carbon nanotubes, have also been explored as ECD electrodes (Radjenovic and Sedlak, 2015). While these materials are easy to synthesize and hold promise as oxygen reduction catalysts (Chen et al., 2017) to form H_2_O_2_, they are often unsuitable for use as anodes in ECD systems as they have low oxygen evolution over-potentials leading to diminished CCS and ^•^OH generation efficiencies compared to BDD.

Despite the promise of BDD, there is limited literature investigating its use as an energy efficient electrode for microbial inactivation. Jeong et al. compared BDD to other anodes and found that it was the most efficient in generating ROS to inactivate *E. coli* and investigated bacterial inactivation using specific ROS (Jeong et al., 2007, 2006; Jeong et al., 2009). It was shown that ^•^OH generated at a constant oxidative current density could disinfect *E. coli* with greater efficiency than CCS and other ROS. However, the mechanism of ECD of blackwater using BDD anodes is unclear. It may be from chlorination, or from production of ^•^OH, H_2_O_2_, ^•^O_2_^−^, and O_3_. Jeong et al. (Jeong et al., 2006; Jeong et al., 2009) attempted to determine the mechanism using multiple disinfection studies by varying the electrolyte and using scavengers. They determined that while chlorination can often be the cause for disinfection when using a BDD anode, the most kinetically favorable electrochemical pathway to producing CCS is likely indirect oxidation of Cl^−^ mediated by ^•^OH. It should be mentioned that these studies did not include reductive generation of H_2_O_2_, which is possible from BDD with surface non-diamond content, such as boron-doped ultrananocrystalline diamond (BD-UNCD) (Thostenson et al., 2017).

ECD of microorganisms has commonly been studied through constant current oxidative methods, although potentiometric methods promise greater coulombic efficiency due to targeted ROS generation (Martínez-Huitle et al., 2015). In constant current methods, the potential of the cell increases with time to maintain the applied current-density, often moving the cell potential well beyond the onset of ROS generation and into the oxygen evolution reaction (OER) and/or hydrogen evolution reaction, sacrificing efficiency for time savings (Martínez-Huitle et al., 2015; Martínez-Huitle and Ferro, 2006). Unlike constant current and potentiostatic methods, potentiodynamic methods provide potentially increased efficiency by keeping the electrode from fouling through reverse polarization and controlling the potential (Macpherson, 2015).

In contrast to other BBD electrodes, BD-UNCD electrodes have a unique ability to generate ROS in aqueous environments at both anodic and cathodic potentials. Recently, our group reported on generation of oxidants and energy efficient disinfection of *E. coli* using BD-UNCD electrodes (Raut et al., 2014, 2013; Thostenson et al., 2017). We previously demonstrated that oxidative functionalization of sp^2^-bonded carbon present on the BD-UNCD surface can catalyze the reductive generation of H_2_O_2_ from dissolved oxygen following the oxygen reduction reaction (ORR) (Thostenson et al., 2017). Subsequent potential cycling was shown to create and stabilize these ORR catalysts through a potentiodynamic-controlled process. Similar correlations of sp^2^ and defective carbon structures have been cited to catalyze the ORR for H_2_O_2_ production (Chen et al., 2017; Macpherson, 2015).

Here we report on the benefits of potential cycling, a potentiodynamic method, for sanitizing blackwater and compare it to potentiostatic methods. We focus on potentiometric operation rather than constant current operation to ensure preservation of catalytic functional groups on the surface of BD-UNCD electrodes by not over-oxidizing or over-reducing them (Thostenson et al., 2017). Moreover, we demonstrate that potential cycling between targeted potentials using functionalized BD-UNCD electrodes in diluted blackwater can decrease the energy needed for disinfection of microbial species. Functionalized BD-UNCD electrodes are shown to provide binding sites for improved electrochemical processes. Subsequent potential cycling of the functionalized BD-UNCD electrodes serves the dual purposes of maintaining the binding sites and keeping the ORR catalysts active. Through a 24 h study in undiluted blackwater, the potential cycling of BD-UNCD is demonstrated to yield an electrode surface with less fouling and with higher current efficiency compared to potentiostatic methods. This work adds to the continuing investigations of ORR catalysts using functionalized carbon materials that have the potential to bring a cost-effective, energy efficient, and practical solution to the problem of disinfecting blackwater.

## 2 Methods

### 2.1 Electrode fabrication

BD-UNCD films (thickness 2 μm) on SiO_x_/Si (1 μm/500 μm) with geometric areas between 0.5 and 1.5 cm^2^ were cleaved from a 4 in (10.16 cm) UL25 wafer purchased from Advanced Diamond Technologies (Romeoville, IL). The wafer has a reported surface roughness of <10 nm rms, with grain sizes on the order of 3–5 nm and electrical resistivity of 0.1 Ω-cm. Cleaved pieces from this wafer were electrically connected to a thin copper wire using silver paste (Ted Pella, PN#16031) and front contact made to the BD-UNCD surface. The contact was left to dry on a hot plate at 80 °C for several hours. The copper wire and paste were then isolated from the electrolyte solution using a glass tube and non-conductive epoxy (Loctite EA 9462 Hysol).

### 2.2 Electrochemical measurements

All measurements were made using a SP-200 Bio-Logic potentiostat. Prior to testing, samples were thoroughly rinsed with deionized water and blown dry with ultra-high purity N_2_ gas (Airgas Inc, PN# NI UHP 300). Unless otherwise noted, all electrochemical measurements were made in a 2-electrode, 100 mL cell where the BD-UNCD wafer and a Pt-wire were the working and counter electrodes, respectively. A 2-electrode cell was used due to space constraints with all indicated potentials being the total cell potential. The positive or negative sign indicates whether BD-UNCD was used as an anode or a cathode, respectively. The Pt counter electrode was also used as a pseudo-reference electrode. In our conditions, its potential remained constant at 0.5 V vs Ag/AgCl in 0.2 M KH_2_PO_4_ with and without tert-butyl alcohol (t-BuOH) and 0.5 M H_2_SO_4_, and 0.4 V vs. Ag/AgCl in 0.154 M NaCl. Further information about use of pseudo-reference electrodes can be found in (Inzelt et al., 2013) and is common when electrolyte conditions may impair standard reference electrodes as is the case when working with blackwater. In our previous publication, the indicated potentials in a 2-electrode cell at pH 0.5 were found to be sufficient to produce H_2_O_2_ via combination of anodic generated ^•^OH or cathodic reduction of dissolved O_2_ as determined through colorimetry (Thostenson et al., 2017). When possible, a 3-electrode system comprised of a standard reference electrode (such as Ag/AgCl) is preferred and enables more precise measurement of the working electrode potential on a standard scale. However, in our cell conditions and when working with blackwater in particular, there are 2 reasons why use a pseudo-reference electrode is preferred. First, particulate matter from blackwater collecting in the frit of a reference electrode may obstruct accurate measurement of the applied potential. Second, disinfection studies using a 2-electrode cell configuration, rather than a 3-electrode cell configuration, highlights the applicability of the described potential cycling methods herein to real-world systems that do not use a 3-electrode configuration. The cell was stirred at a constant 350 rpm throughout the measurements to improve mixing of generated oxidants and microbial species. A schematic of the cell can be found in SM Fig. 1. Details regarding a control experiment to validate use of the Pt-wire as a pseudo-reference electrode can be found in the Supplementary Material including SM Fig. 2. Unless otherwise noted, BD-UNCD was anodized at + 2 V in 0.5 M H_2_SO_4_ for 20 min prior to each measurement.

**Fig. 1:**
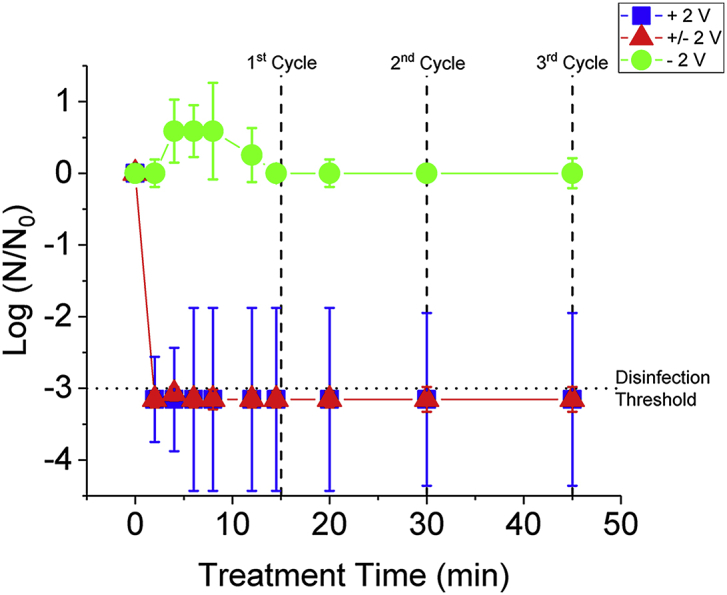
Bacterial inactivation from 3 methods of potentiometric testing. Two potentiostatic methods (+ 2 V and - 2 V) were compared to a potentiodynamic method ( ± 2 V) in blackwater diluted 1:501 with 0.154 M NaCl. The error bars are the standard deviation from 3 trials. Dashed lines indicate the beginning of the indicated potentiodynamic cycle for the ± 2 V treatment and the horizontal dotted line indicates the STeP disinfection threshold (<5 MPN mL^−1^).

**Fig. 2:**
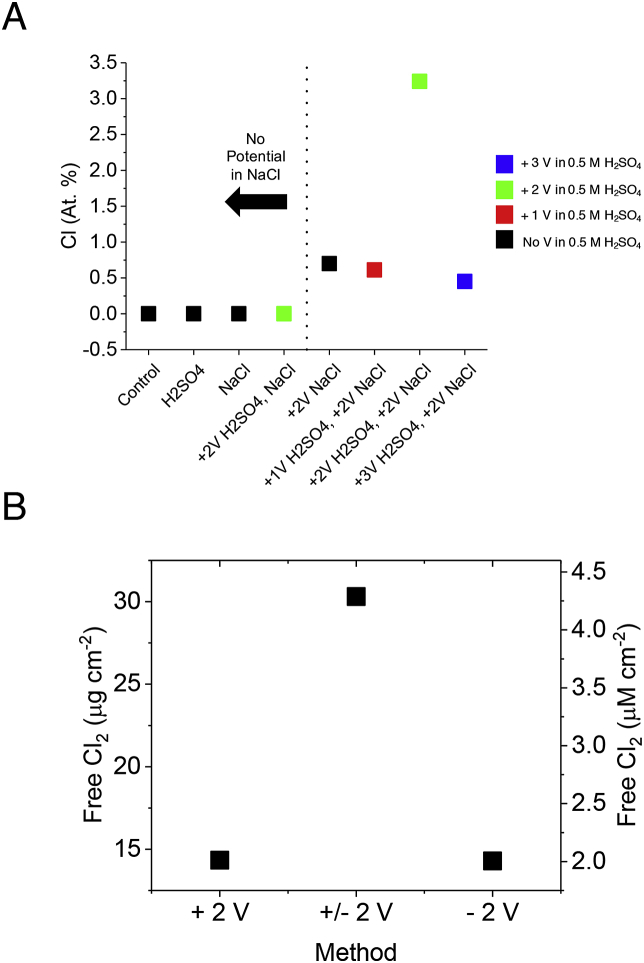
A) Chlorine containing species (CCS) adsorbed on BD-UNCD electrodes as a function of pre-treatment conditions. Where no voltage is indicated, the electrode was left in the indicated electrolyte without bias for the same amount of time as those electrodes that were polarized (20 min in 0.5 M H_2_SO_4_ and 45 min in 0.154 NaCl). The color of each point indicates the magnitude of anodic polarization in 0.5 M H_2_SO_4_. B) Free chlorine generation versus treatment method after 45 min electrolysis in 0.154 M NaCl.

### 2.3 Diluted blackwater experiments

Diluted blackwater experiments discussed here were the result of 3 experiments per treatment method per electrolyte solution.

Blackwater Collection and Use: Blackwater was collected from a prototype toilet system that processes human waste at RTI International. The details of the toilet prototype and composition of blackwater have been described previously (Hawkins et al., 2017; Sellgren et al., 2017). Blackwater was used within 3 days of its collection to stay within the 1 week time-frame that bacterial concentrations were found to be stable in ambient lab conditions.

Dilution of Blackwater in Electrolytes: To shorten the timescale of the experiments and decrease electrode size, blackwater was diluted 1:501 in 3 different supporting electrolyte solutions, with each chosen for controlled study of different oxidizing species. This was done by adding 1 mL of well-stirred blackwater to 500 mL of the bulk electrolyte. The solution was then stirred for several minutes and bubbled with ultra-high purity O_2_ (Airgas) for 10 min prior to the experiment to ensure oxygen saturation in the solution. The 3 electrolyte solutions used were 0.154 M NaCl (Sigma Aldrich), 0.2 M KH_2_PO_4_ (Sigma Aldrich), and 0.05 M t-BuOH (Alfa Aesar) in 0.2 M KH_2_PO_4_. The solvent water used was dispensed from a Milipore Q water system. In a control study, these solutions were found to have no effect on bacterial stability outside of electrochemical experiments. All solutions using diluted blackwater were found to have a pH 7.

Experimental Procedure: A static (+ 2 V or – 2 V) or dynamic (+ 2 V followed by – 2 V) potential was applied to a BD-UNCD electrode with respect to a Pt-wire in diluted blackwater solutions for 45 min. The same electrode was used across treatment methods. When the electrolyte solution used in the blackwater dilution was changed, a new electrode was then used. This was done to allow for equal comparison of each treatment method while also limiting the complications of electrode history when changing electrolytes. For the potentiodynamic treatment method, + 2 V was held first for 13 min 20 s followed by – 2 V for 1 min 40 s to complete a 15 min cycle. This method was optimized to more efficiently produce H_2_O_2_ compared to potentiostatic methods in our previous publication (Thostenson et al., 2017). Three total cycles were completed within the 45 min treatment time-frame. The Pt-wire was separated from the cell by a nylon membrane (0.2 μm, Sigma Aldrich #58060-U) to limit the effects of Pt on disinfection. The cell was continuously stirred at 350 rpm to ensure mixing of microbial pathogens and oxidizing species. 1 mL samples of the diluted solution were taken to measure bacterial concentrations prior to treatment (t = 0) and at t = 2, 4, 6, 8, 12, 14.5, 20, 30 and 45 min. Most probable number (MPN) measurements of the samples were then made within 12 h using refrigerated samples (Blodgett, 2010). Lysogeny broth (LB) agar substrates were used to obtain a total (non-specific) bacterial count from the MPN assay. When refrigerated, 1 mL samples were found to have stable bacterial concentrations for several days. Free chlorine was measured using N,N Diethyl-1,4 Phenylenediamine Sulfate (DPD) (HACH method 8167) and a HACH DR 900 colorimeter (HACH, Loveland, CO). Samples were run per the manufacturer's instructions. Blanks consisting of the diluted blackwater and electrolyte solutions without the addition of the DPD reagent were run before each measurement with no free chlorine found.

### 2.4 Microbial enumeration

Microbial enumeration measurements using MPN were made according to Sellgren et al. (Sellgren et al., 2017). Reported values are the average of 3 trials and normalized to the initial MPN, with each independent trial presented in Supplementary Material (SM Fig. 4, Fig. 5, Fig. 6). The bars at each data point indicate the standard deviation of the 3 measurements. Reported values for energy required to disinfect were taken as the average of 3 measurements and indicate the energy used to reach the Sanitation Technology Platform (STeP) disinfection threshold (<5 MPN mL^−1^), or the resultant bacterial concentration at the end of each measurement in the case that the STeP disinfection threshold was not reached. These values are shown normalized to the cell suspension volume (100 mL) and log reduction of bacteria for comparison between electrolyte solution and experiment.

### 2.5 X-ray photoelectron spectroscopy (XPS)

Collection: X-ray photoelectron spectroscopy (XPS) was conducted using a Kratos Analytical Axis Ultra instrument with a monochromated Al K_α_ X-ray source (1486.69 eV) operated at 15 kV and 10 mA (150 W) in a 5 × 10^−8^ Torr chamber. Survey spectra were collected using an analyzer pass energy of 160 eV, and binding energies were collected from − 5 to 1200 eV scanned at 1 eV increments. Each step was integrated for 500 ms and the entire spectrum was averaged across 3 sweeps.

Analysis: Collected spectra were calibrated and analyzed using Casa XPS software following best practices as outlined in Briggs and Grant. (Briggs and Grant, 2003). All data reported were the average of 3 measurements per sample. The XPS Cl at. % values were determined by normalizing the integrated Cl 2p peak intensity centered at a binding energy of 200 eV to the integrated C 1s and O 1s peak intensity centered at 285 eV and 532 eV, respectively. Shirley background subtraction was used for all peaks.

### 2.6 Undiluted blackwater experiments

Unless otherwise mentioned, procedures described in Section 2.3 were followed for the undiluted blackwater experiments. Three BD-UNCD electrodes were held at + 2 V, −2 V, or switched between these two potentials ( ± 2 V treatment method) versus a Pt wire counter electrode for 24 h in a 20 mL cell of blackwater. The cell was stirred at 350 rpm throughout the 24 h. Change in current density measurements reported for each electrode were made at the first 100 s and last 5 s of treatment (23:59:55 of the format HH:MM:SS) for the + 2 V and – 2 V methods. This measurement was used to signify a decrease in electrode performance resulting from electrode fouling or catalyst depletion. For the ± 2 V treatment method, the change in anodic current density was taken as the difference between current density measured at 100 s and at 5 s before the last reduction half-cycle (23:58:15). The change in cathodic current density was taken as the difference between the current measured at 100 s after the start of the first cathodic half-cycle (00:15:00), which was just before the start of the 2nd potentiodynamic cycle and at 5 s before the end of 24-h treatment (23:59:55). Further information regarding the selection of these time points can be found in Supplementary Material.

## 3 Results

### 3.1 Comparison of potentiostatic and potentiodynamic methods in the disinfection of diluted blackwater

Generation of reactive oxygen species (ROS) and chlorine containing species (CCS) by potentiostatic (+ 2 V and − 2 V) and potentiodynamic ( ± 2 V) methods were compared with different electrolyte solutions as summarized in Table 1. The effect of electrolyte solution, generation of CCS and ROS from BDD and microbial inactivation mechanisms was previously described by Jeong et al. (Jeong et al., 2006; Jeong et al., 2009), but will now be briefly summarized. NaCl (0.154 M) electrolyte solution allowed study of disinfection of blackwater when Cl^−^, H_2_O, and O_2_ were present in solution to create CCS and ROS. In this case, microbial inactivation was caused by direct redox, CCS, and ROS. KH_2_PO_4_ (0.2 M) provided the similar conductivity as 0.154 M NaCl but allowed study of disinfection of blackwater without addition of Cl^−^, other than that naturally contained in urine which is ∼5.6 g L^−1^ NaCl (Putman, 1971), leaving only reactants for ROS (H_2_O and O_2_) present. In this case, microbial inactivation was accomplished by direct redox and ROS. Addition of t-BuOH (0.05 M) to the KH_2_PO_4_ solution allowed for the study of disinfection in the absence of Cl^−^ and ^•^OH. In this case, t-BuOH scavenged ^•^OH such that microbial inactivation happened from direct redox and ROS while the effect of ^•^OH oxidation of microbial species was largely suppressed. Each of these cases are discussed in more detail below.

**Table 1:** Summary of diluted blackwater treatment methods and expected oxidant species generation in different electrolytes.

Method	Electrolyte	Generated oxidants
+ 2 V	NaCl	CCS + ROS
	KH_2_PO_4_	ROS
	KH_2_PO_4_ + t-BuOH	
- 2 V	NaCl	H_2_O_2_
	KH_2_PO_4_	H_2_O_2_
	KH_2_PO_4_ + t-BuOH	H_2_O_2_
± 2 V	NaCl	CCS + ROS
	KH_2_PO_4_	ROS
	KH_2_PO_4_ + t-BuOH	H_2_O_2_

#### 3.1.1 Disinfection in chloride containing electrolytes

Fig. 1 shows the bacterial inactivation of diluted blackwater in 0.154 M NaCl. The – 2 V treatment has little impact on the reduction of bacterial concentration in the diluted blackwater. Since the – 2 V electrode is only able to generate ROS, such as H_2_O_2_ and ^•^O_2_^−^ from dissolved O_2_, it is likely that the concentration of dissolved oxygen in solution is too low and that O_2_ absorption rate in the setup tested is too slow to result in significant disinfection. The overpotential of the – 2 V treatment was well beyond the onset of the ORR (found to be ∼ - 0.7 V vs. Ag/AgCl in our conditions), so it is unlikely the absence of disinfection was due to insufficient overpotential. Still, it may be related to diminished electrode kinetics since BD-UNCD is a poor ORR electrode when the non-diamond content at the surface is not appropriately functionalized.

For the + 2 V and ± 2 V treatments, the disinfection was rapid, both reducing bacterial concentrations by 3 orders of magnitude in the first 2–6 min of treatment. The disinfection required little energy per log reduction of microbial concentration with both the + 2 V and ± 2 V treatment methods using only 57 ((μW h)/(L log(N_0_/N))). From our previous work (Thostenson et al., 2017), pre-treatment of BD-UNCD electrodes in 0.5 M H_2_SO_4_ was hypothesized to have been responsible for the rapid and energy efficient disinfection of the anodic methods by improving the generation of disinfecting CCS seen in Fig. 1. Reaction pathways of forming disinfecting CCS from reaction of ^•^OH with CCS from BDD anodes is well reported in literature with a great overview of the potential reaction pathways presented in Polcaro et al. (Polcaro et al., 2009). As described in Polcaro et al. (Polcaro et al., 2009), BDD anodes in chloride containing electrolytes can generate HClO directly, or can generate ClO_2_, ClO_3_^−^, ROS by secondary reaction with ^•^OH. All of the species are strong oxidizers and disinfecting species. To test the hypothesis that pre-treatment of BD-UNCD can improve the generation of CCS, different pre-treatments of BD-UNCD electrodes were made and the resultant chlorine content on each electrode was measured using XPS to indirectly quantify the adsorbed CCS content present on the surface. It was found that CCS are only present on the electrode when a positive voltage is applied to the BD-UNCD electrodes in a 0.154 M NaCl solution (Fig. 2A). Pre-treatment of BD-UNCD in 0.5 M H_2_SO_4_ for 20 min at anodic potentials was found to further increase CCS adsorption in 0.154 M NaCl by partially oxidizing surface sp^2^ carbon bonds, leading to functional sites at the surface of BD-UNCD electrodes that serve as adsorption sites. As previously mentioned, BD-UNCD has a significant amount of surface sp^2^-bonded carbon. BD-UNCD that is free of non-diamond content (*i.e.*, sp^2^) is effectively unable to adsorb surface chemical species (Macpherson, 2015; Patten et al., 2012). Proper oxidation at + 2 V vs. Pt of the non-diamond surface carbon can create an optimal concentration of oxygenated functional sites. We previously showed how such functional groups can be created and maintained for catalysis of the ORR (Thostenson et al., 2017). As seen in Fig. 2A, a lesser or greater potential than + 2 V vs. Pt diminishes this adsorption effect. This is likely due to under-oxidation of the non-diamond carbon in the 1 V experiments and removal of the non-diamond carbon in the 3 V experiments (Ayres et al., 2016; Hutton et al., 2013; Thostenson et al., 2017). On the one hand, under-oxidation (<2 V vs. Pt-wire) of non-diamond carbon on BD-UNCD electrode surfaces diminishes adsorption by leaving the surface in its native H-terminated surface which is hydrophobic (Macpherson, 2015). On the other hand, over-oxidation (>2 V vs. Pt-wire) of the non-diamond carbon will create an O-terminated surface that is hydrophilic, but has less-conductive double-bonded carbon-oxygen functional groups at the surface or has removed the non-diamond carbon entirely (Bennett et al., 2004; Macpherson, 2015; Williams, 2011). In both 1 V and 3 V experiments, this results in diminished CCS adsorption compared to the 2 V because there is a lesser concentration of appropriately oxygenated functional groups on the BD-UNCD electrode surface.

Further study revealed that potential cycling significantly increased the generated concentration of CCS by maintaining an increased concentration of adsorbed Cl^−^ on the BD-UNCD surface. This is shown in Fig. 2B where the ± 2 V potential cycling method produced more free chlorine per electrode area than the +2 V and – 2 V potentiostatic methods. The increased free chlorine generation from the ± 2 V method indicated that reverse polarization and continued regeneration of the functional groups significantly increased the generation of CCS. As stated in the methods section, no residual chlorine was found in solution prior to electrolysis. Moreover, the pH for all diluted blackwater solutions was pH 7 so volatilization of free-chlorine should be minimal as has been reported previously in literature (Schaefer et al., 2017).

**Fig. 3:**
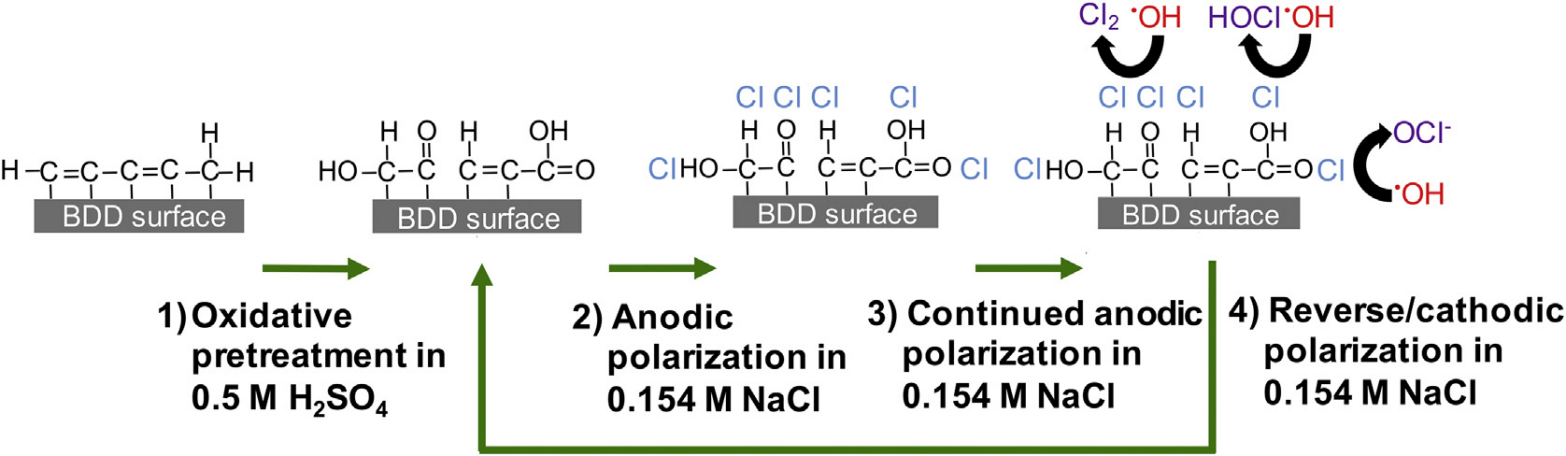
Proposed surface chemical processes of BD-UNCD electrode with non-diamond content on surface. Step 1) oxidation in 0.5 M H_2_SO_4_ at + 2 V leads to oxygenated functional groups at surface. Step 2) anodic polarization in 0.154 M NaCl leads to Cl^−^ adsorption on to functional groups. Step 3) Continued anodic polarization in water leads to oxidation of adsorbed Cl^−^ from generated ^•^OH and formation of CCS. Step 4) Reverse/cathodic polarization in H_2_O desorbs CCS content for dispersal into solution and regenerates non-diamond functional groups on BD-UNCD surface.

A proposed mechanism of increased CCS generation resulting from appropriate pre-treatment and potential cycling is shown in Fig. 3. Pre-treatment of the BD-UNCD electrode surface in 0.5 M H_2_SO_4_ plays a vital role in how well Cl^−^ adsorbs, summarized as follows. Step 1: Oxidation in H_2_SO_4_ creates oxygenated functional groups on non-diamond content on the BD-UNCD surface (Thostenson et al., 2017). Step 2: Anodic polarization of the functionalized electrode in Cl^−^-containing solutions (in this work 0.154 M NaCl) leads to the adsorption of Cl^−^ anions on the oxygenated functional groups created in Step 1. Step 3: Continued anodic polarization at sufficient over-potentials in aqueous solutions will generate ^•^OH that reacts with the adsorbed Cl^−^, yielding CCS. Step 4: Reverse polarization to a cathodic potential discharges CCS from the electrode surface and reactivates oxygenated functional group catalysts.

**Fig. 4:**
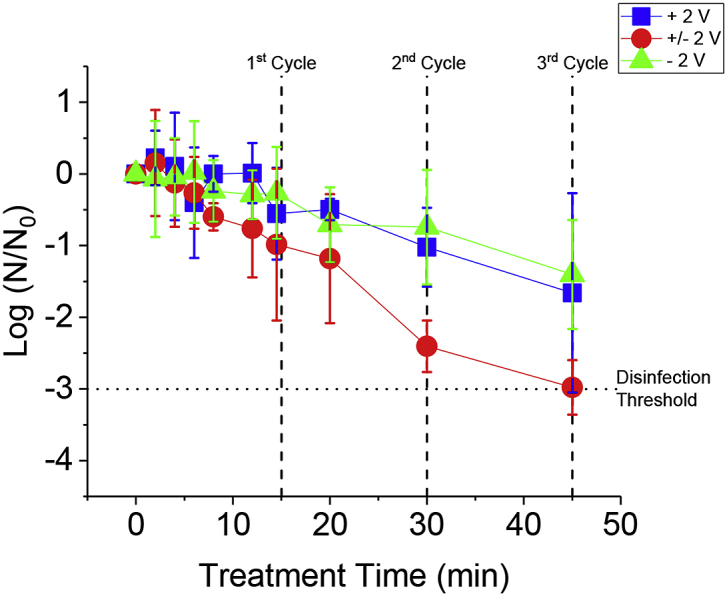
Disinfection of diluted blackwater in 0.2 M KH_2_PO_4_ (1:501) using 3 different treatment methods. The error bars are the standard deviation from 3 trials. The vertical dashed lines indicate the end of the indicated potentiodynamic cycle for the ± 2 V treatment. The horizontal dotted line indicates the STeP threshold of disinfection (<5 MPN mL^−1^).

**Fig. 5:**
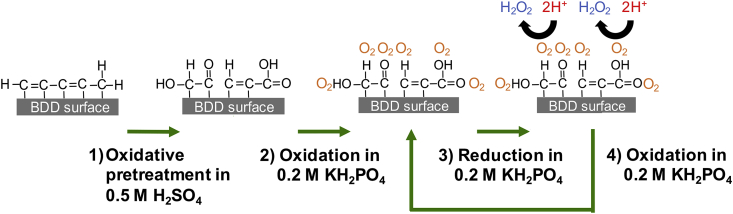
Proposed Electrochemical Advanced Oxidation Process (EAOP) from potential cycling. Step 1) Oxidation in 0.5 M H_2_SO_4_ at + 2 V leads to oxygenated functional groups at surface. Step 2) Oxidation in 0.2 M KH_2_PO_4_ at + 2 V leads to water splitting and with O_2_ adsorption on to functional groups. Step 3) Reverse polarization and subsequent reduction in water leads to reduction of adsorbed O_2_ and formation of H_2_O_2_. Step 4) Reverse/anodic polarization in H_2_O regenerates non-diamond functional groups on BD-UNCD surface and evolves O_2_ from water oxidation that adsorbs again onto the surface beginning the process again following step 2.

**Fig. 6:**
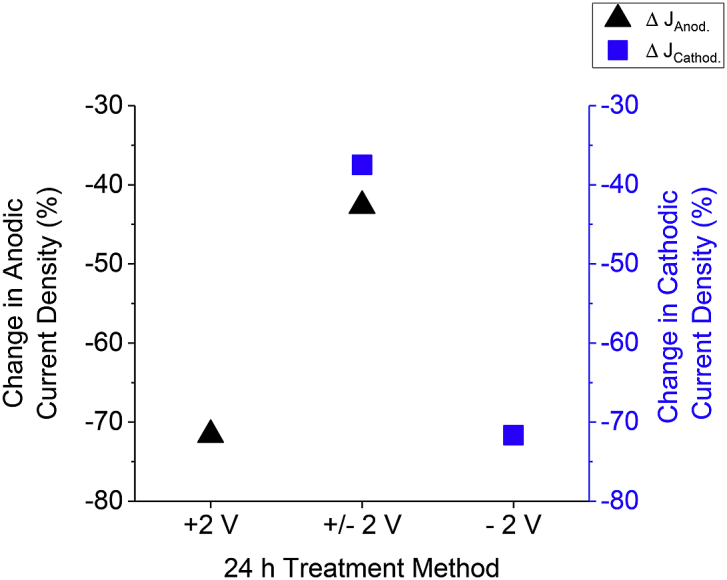
Change in anodic (triangles) and cathodic (squares) current densities of the indicated potentiometric treatment from 24 h electrolysis of undiluted blackwater. The + 2 V and – 2 V treatment methods were potentiostatic while the ± 2 V treatment method was potentiodynamic (1 cycle was + 2 V for 13 m 20 s and – 2 V for 1 m 40 s, and there were 96 cycles made in 24 h).

Since ^•^OH radicals are short lived with very short diffusion lengths, the generation rate of CCS is much higher with Cl^−^ ions in close proximity and high concentration near the electrode surface than if Cl^−^ ions were not adsorbed (*i.e.*, dispersed in solution). BD-UNCD without appropriately functionalized non-diamond carbon on the surface is unable to adsorb Cl^−^ due to lack of adsorption sites. This is evidenced by the + 3 V H_2_SO_4_, + 2 V NaCl method in Fig. 2A. Potential cycling, more specifically reverse polarization, increases CCS generation as shown in Step 4 of Fig. 3. There are two possible reasons for the increased CCS generation. First, adsorbed CCS content that is discharged from the electrode surface during the negative half-cycle of the potential cycling method. The CCS are then dispersed into solution, leading to a higher free-chlorine concentration. Second, reverse polarization of the BD-UNCD electrode surface regenerates the functionalized non-diamond carbon catalyst for increased free-chlorine generation.

While these results were promising, the data gave neither direct insight into the role of ROS in disinfection of blackwater, nor did they highlight key differences between potentiostatic and potentiodynamic methods. Since there was a significant concentration of CCS generated and disinfection was rapid, comparison with a Cl^−^ free electrolyte solution was needed to give mechanistic insight. Moreover, the time-scale of disinfection was too rapid to distinguish potentiostatic and potentiodynamic methods. By removing Cl^−^, the role of ROS on disinfection from potentionstatic versus potentiodynamic methods could be more directly studied.

#### 3.1.2 Disinfection in chloride free electrolytes

To determine the effect of ROS generated and their rate from the potentiodynamic compared to potentiostatic methods, KH_2_PO_4_ was employed to provide a chlorine free electrolyte solution. Fig. 4 shows the disinfection of diluted blackwater in 0.2 M KH_2_PO_4_. As can be seen in Fig. 4, the potentiodynamic method ( ± 2 V, red circles) was able to reach the Sanitation Technology Platform (STeP) disinfection threshold and decrease microbial species concentration by 1.5 orders of magnitude below the potentiostatic method (+ 2 V, blue squares and – 2 V, green triangles) during the 45 min treatment. At + 2 V in an aqueous electrolyte, ^•^OH is generated by a BD-UNCD electrode, which can lead to indirect generation of other ROS, such as H_2_O_2_, O_3_, and ^•^O_2_^−^ (Jeong et al., 2006). These second order ROS were considered responsible for inactivation of microbial species in the + 2 V and ± 2 V treatment methods. Jeong et al. demonstrated that H_2_O_2_ is the only second order ROS generated from BDD at anodic potentials to have an impact on bacterial reduction (Jeong et al., 2006; Jeong et al., 2009). At – 2 V, there is only the generation of H_2_O_2_ via reduction of dissolved oxygen. In the ± 2 V treatment method, H_2_O_2_ is not only generated at a cathodic potential, but also at an anodic potential via combination of ^•^OH. The + 2 V treatment method also generates H_2_O_2_ at an anodic potential through combination of ^•^OH, but does not reduce dissolved oxygen for formation of H_2_O_2_. Despite the different reaction pathways in generating H_2_O_2_, the potentiostatic methods (+ 2 V and – 2 V) seen in Fig. 4 resulted in very similar disinfection curves, suggesting H_2_O_2_ was largely responsible for the disinfection as it was the only ROS shared by the two methods. The impact of H_2_O_2_ on disinfection is further demonstrated by the ± 2 V treatment method which both reaches the disinfection threshold (Fig. 4) and does so more efficiently (Table 2) than the other methods. Among other reasons described in the proceeding paragraphs, the generation of H_2_O_2_ from the ± 2 V treatment method was previously shown to be higher and more efficient than the other static methods (+ 2 V and – 2 V) with coulombic efficiencies approaching 23% and 50% for the + 2 V and – 2 V potentials, respectively (Thostenson et al., 2017). Conversely and under different conditions, Jeong et al. have shown that ^•^OH is likely responsible for the disinfection of *E. coli* when using anodic potentials on BDD (Jeong et al., 2009; Jeong et al., 2006), and ruled out the contributions of H_2_O_2_ from BDD at anodic potentials by adding H_2_O_2_ to an *E. coli* solution as part of a control experiment. Since blackwater was treated instead of *E. coli* in this study, this difference as well as other cell conditions that differ with Jeong et al., could explain why we found H_2_O_2_ to be an effective oxidizer of microbial species.

**Table 2:** Energy for bacterial reduction for the + 2 V, - 2 V, and ± 2 V treatment methods. Energy is given normalized to the logarithmic magnitude of bacterial reduction and cell volume. These figures of merit indicate the efficient utilization of energy of each particular treatment method in 1 L of each respective electrolyte for the remediation of 1 order of magnitude of microbial species.

METHOD	Energy for bacterial reduction in 0.2 M KH_2_PO_4_ (μW h/(L log(N_0_/N)))	Energy for bacterial reduction in 0.2 M KH_2_PO_4_ + 0.05 M t-BuOH (μW h/(L log(N_0_/N)))
+ 2 V	325	4860
+/- 2 V	262	3450
- 2 V	588	NA

The potentiodynamic method ( ± 2 V, red circles) seen in Fig. 4 shows improved disinfection over the potentiostatic treatment methods for three possible reasons. First, potential cycling leads to the continuous charging and discharging of the electrode-electrolyte double layer. This leads to an increased number of adsorbed species on the electrode surface over time, which can result in a higher probability of direct reduction-oxidation (redox) of microbial species and generation of oxidants compared to potentiostatic methods. Second, the anodic over-potential in water creates O_2_ in high concentration at the electrode surface. This O_2_ is likely to adsorb at non-diamond carbon functional groups created by oxidative pre-treatment in 0.5 M H_2_SO_4_. When the electrode is cycled to a reducing potential, the O_2_ is readily available at the electrode for reduction to H_2_O_2_ following the oxygen reduction reaction. This process is similar to that proposed in Thostenson *et al.* (Thostenson et al. (2017) and to that of the electrochemical advanced oxidation process (EAOP) which require O_2_ injected at the electrode surface for reduction to H_2_O_2_ at higher efficiencies (Martínez-Huitle and Ferro, 2006). Third, potential cycling of BD-UNCD between – 2 V and + 2 V vs. Pt-wire creates and maintains catalytic functional groups for the reductive generation of H_2_O_2_ from dissolved oxygen. We previously described this catalytic process (Thostenson et al., 2017).

A proposed new EAOP process from potential cycling of BD-UNCD in water based electrolytes is illustrated in Fig. 5. Diamond does not have the binding sites needed for the ORR, while non-diamond and functionalized carbon do, as indicated by their adsorption properties (Macpherson, 2015). Pre-treatment of the non-diamond carbon in 0.5 M H_2_SO_4_ at + 2 V functionalizes the non-diamond carbon present at the BD-UNCD surface (Step 1, Fig. 5). Proper functionalization of the BD-UNCD surface was shown earlier in this paper to improve adsorption of Cl^−^ and subsequently improve CCS generation. In the case of a Cl^−^ free electrolyte, the functional groups adsorb O_2_ rather than Cl^−^ at + 2 V (Step 2, Fig. 5). Cathodic polarization at – 2 V in H_2_O reduces the O_2_ to form H_2_O_2_ (Step 3, Fig. 5). Applying an anodic potential at + 2 V again generates oxygen that adsorbs onto the functional groups. As described previously (Thostenson et al., 2017), oxygenated functional groups occurring on non-diamond carbon (such as sp^2^ bonded carbon on the surface of BD-UNCD) provide enhanced oxygen reduction kinetics.

The energy savings resulting from this EAOP process in Cl^−^ free electrolyte solutions can be seen in Table 2, which reports the energy required to disinfect diluted blackwater. Potential cycling has a net energy savings of 24% and 124% compared to the + 2 V and – 2 V treatment methods, respectively, when ^•^OH is present (Table 2, middle column).

To determine what effect ^•^OH had on disinfection, 0.05 M t-BuOH was added to a 1:501 dilution of blackwater in 0.2 M KH_2_PO_4._ t-BuOH is a well-known ^•^OH scavenger that will quickly react with ^•^OH in solution to mitigate the role of ^•^OH in disinfection (Jeong et al., 2009; Jeong et al., 2006). Table 2 (right column) demonstrate the detriment of removing ^•^OH as a disinfecting ROS. The 3 treatment methods showed diminished inactivation efficiency of microbial species in diluted blackwater. This result indicates a strong dependence on the anodic generation of ROS and their dispersal into solution for disinfection. There are two reasons this is the case. First, anodically generated ROS such as ^•^OH or the further reaction of ^•^OH to form H_2_O_2_, O_3_ and ^•^O_2_^−^ is largely suppressed by the t-BuOH which will scavenge ^•^OH. This results in a diminished concentration of ROS in the bulk solution for disinfection of microbial species. In addition, scavenging ^•^OH likely inhibits the oxidation of functional groups on the BD-UNCD surface (Fig. 5, Steps 2 and 4), which is important to the effectiveness of the ± 2 V potential cycling method. As previously discussed, oxygenated functional groups serve as adsorption sites for O_2_ and catalysts for O_2_ reduction to H_2_O_2_ upon cathodic polarization (Fig. 5, Step 3). Second, direct redox of microbial species is a small part of the overall disinfection seen in Fig. 1, Fig. 4. Direct redox is the only uninhibited inactivation mechanism when t-BuOH was added to the KH_2_PO_4_ and blackwater solution. Table 2 (right column) indicates that removing ^•^OH as a direct oxidant of microbial species has a detrimental effect on disinfection energy efficiency and largely limits the ability to disinfect blackwater (as can also be seen in SM Fig. 6). Taken together, these data support the conclusion of Jeong et al. (Jeong et al., 2006; Jeong et al., 2009) that bacterial inactivation by ^•^OH is energy efficient and effective.

### 3.2 Comparison of electrode fouling effects from treatment methods in undiluted blackwater

The potentiostatic and potentiodynamic methods were used to treat undiluted blackwater for 24 h so that their performance over time could be compared. Potentiostatic electrolysis commonly leads to diminished electrode current densities due to the accumulation of a resistive film on the electrode surface, known as fouling (Schmalz et al., 2009). Through reverse polarization, the film can be removed and the electrode returned to its original current density. BDD electrodes have been cited to be stable under reverse polarization (Macpherson, 2015; Vinokur et al., 1996; Williams, 2011). Following 24 h electrolysis, no reduction in microbial species was measured (See SM Fig. 7 for disinfection curves) likely resultant from high concentrations of chemical oxygen demand and the electrolysis period being too short. However, differences in current densities before and after 24 h electrolysis between the treatment methods were found and are summarized in Fig. 6. As shown in Fig. 6, the potentiostatic (+ 2 V and – 2 V) methods result in current densities at the end of the treatment that are roughly 70% less than their initial steady state current density. The anodic and cathodic current density of the ± 2 V treatment decreases only 40% during the 24 h treatment, much less than the potentiostatic methods. The difference between the potentiostatic methods compared to the potentiodynamic method lies in the fact that the continued potential cycling of the of the ± 2 V treatment reverse polarizes the electrode and discharges the accumulated film. The higher anodic and cathodic current densities for a given time and potential means improved electrolysis efficiency compared to the potentiostatic treatments. As mentioned before, these energy saving benefits are important to ECD of blackwater. Moreover, frequently servicing an electrode in an operating environment can be problematic and impractical. Minimizing fouling of an electrode surface decreases electrode maintenance and allows for a more cost-effective and long-term solution. Thus, the proposed potentiodynamic method has direct benefits to improving ECD of blackwater across the evolving field of decentralized wastewater treatment.

## 4 Conclusions

Here we report on the benefits of potential cycling, a potentiodynamic method, in sanitizing blackwater, and compared it to two potentiostatic methods. It was demonstrated that potential cycling of functionalized BD-UNCD electrodes in diluted blackwater can save on energy expenditure required for disinfection of microbial species. Functionalization of BD-UNCD electrodes through potential cycling is shown to provide binding sites for improved electrochemical processes. Subsequent potential cycling of the functionalized BD-UNCD electrodes serves the dual purpose of maintaining these binding sites, which can become fouled with time, as well as keeping their catalytic properties active. Through a 24 h study in undiluted blackwater, the potential cycling of BD-UNCD is demonstrated to yield a more energy efficient disinfection process compared to two other potentiostatic methods. This work adds to the continuing investigations of oxygen reduction reaction (ORR) catalysts and electrochemical advanced oxidation processes (EAOP) using functionalized carbon materials that have the potential to bring a cost-effective, energy efficient, and practical solution to the problem of treating blackwater for decentralized wastewater treatment.
