# Supplementary material for: Improved blackwater disinfection using potentiodynamic methods with oxidized boron-doped diamond electrodes
Source: Water Res. 2018 Sep 1;140:191–9. doi: 10.1016/j.watres.2018.04.022 (PMC5995412; doi:10.1016/j.watres.2018.04.022)
Supplement: Online data [file mmc1.docx]

Supplementary Material

Improved Blackwater Disinfection Using Potentiodynamic Methods with Oxidized Boron-Doped Diamond Electrodes

Thostenson, J. O.^1,2^, Mourouvin, R.^1,3^, Hawkins, B. T.^1,2,4^, Ngaboyamahina, E.^1,2^, Sellgren, K. L.^1,2,4^, Parker, C. B.^1,2^, Deshusses, M. A.^4^, Stoner, B. R.^1,2,4^ and Glass, J. T.^1,2,^^

**Author Affiliations:**

^1^ Department of Electrical and Computer Engineering, Duke University, Durham, NC

^2^ Center for WaSH-AID, Duke University, Durham, NC

^3^ École Centrale de Lyon, Ecully, France

^4^ Research Triangle Institute (RTI) International, Research Triangle Park, NC

^5^ Department of Civil and Environmental Engineering, Duke University, Durham, NC

**^Corresponding Author:**

Jeffrey T. Glass, PhD.

Department of Electrical and Computer Engineering

Duke University

Durham, NC 27708

jeff.glass@duke.edu

# Experimental Cell Configuration and Validation of Pt-Wire Pseudo Reference Electrode

SM Figure 1: Schematic of cell configuration used for electrochemical disinfection experiments

During the + 2 V potentiostatic treatment method, a current of - 150 µA passes through a Pt-wire pseudo-reference electrode. In a control experiment, - 150 µA was applied to the same Pt-wire in a 3-electrode cell configuration while the potential was measured. When steady state is reached after 2 min (see SM Figure 2A), the potential remains stable and constant around - 0.84 V vs. Ag/AgCl. The slight potential fluctuation (+/- 2 mV) observed (SM Figure 2A inset) is likely to arise from hydrogen gas evolution. In a similar manner, 40 µA was applied to the Pt-wire in a 3-electrode measurement to simulate the – 2 V experiment. The potential stabilizes around 1.150 V after 2 min (SM Figure 2B. These experiments indicate that the Pt-wire electrode can maintain a steady potential when small current densities are applied, and therefore can be used as a pseudo-reference electrode in our cell conditions.

SM Figure 2: Usage of Pt-wire as a pseudo-reference electrode validation study with Pt-wire under A) -150 µA and B) 40 µA corresponding to the current produced by the Pt-wire pseudo-reference electrode during the + 2 V and – 2 V potentiostatic treatment methods, respectively. Inset in A shows minor (+/- 2 mV) fluctuations of current during electrolysis. Measurements were made in a 275 mL 0.124 M NaCl 3-electrode cell, with a Ag/AgCl (saturated KCl) reference electrode and Pt-mesh counter electrode.

# Measurement Methodology of Change in Current Density for 24 hr Electrolysis in Undiluted Blackwater

Points in time during a 24 hr experiment in undiluted blackwater were chosen to measure how the anodic and cathodic current densities of the BD-UNCD electrodes decreased over time. The first time-point was chosen at 100 s to minimize the effect that double-layer charging might have on the steady-state current density of the + 2 V, - 2 V and +/- 2 V treatment methods and also to enable proper comparison of changes in current density between these treatment methods. In the case of the +/- 2 V treatment method, the anodic half-cycle lasted 800 s, and the cathodic half-cycle lasted 100 s. Thus, 100 s after the initial applied potential was chosen for appropriate comparison between all methods. The last time-point, 5 s before the last cathodic half-cycle, was chosen to give the ending steady-state current of all treatment methods and their respective anodic or cathodic current densities.

# XPS Spectra

SM Figure 3: A) Survey and B) Cl 2p regional spectra of the XPS data taken of the BD-UNCD electrodes pretreated in 0.5 M H2SO4 at different voltages before than being polarized in 0.124 M NaCl at + 2 V. The indicated potentials are cell potentials vs. a Pt-wire. The spectra have been background subtracted.

# Additional Bacterial Inactivation Measurements

## Diluted Blackwater

### Individual 0.124 M NaCl Disinfection Curves

SM Figure 4: Individual disinfection treatments of diluted blackwater in 0.124 M NaCl (1:501)

### Individual 0.2 M KH_2_PO_4_ Disinfection Curves

SM Figure 5: Individual disinfection treatments of diluted blackwater in 0.2 M KH_2_PO_4_ (1:501)

### Other 0.2 M KH_2_PO_4_ + 0.05 M t-BuOH Disinfection Curves

SM Figure 6: Individual disinfection treatments of diluted blackwater in 0.2 M KH2PO4 + 0.05 M t-BuOH (1:501)

## Undiluted Blackwater

SM Figure 7: 24-hour treatment of undiluted blackwater.
